# Furin and TMPRSS2 Resistant Spike Induces Robust Humoral and Cellular Immunity Against SARS-CoV-2 Lethal Infection

**DOI:** 10.3389/fimmu.2022.872047

**Published:** 2022-05-02

**Authors:** Jhe-Jhih Lin, Chih-Feng Tien, Yi-Ping Kuo, En-Ju Lin, Wei-Hsiang Tsai, Ming-Yu Chen, Pei-Ju Tsai, Yu-Wen Su, Nikhil Pathak, Jinn-Moon Yang, Chia-Yi Yu, Zih-Shiuan Chuang, Han-Chieh Wu, Wan-Ting Tsai, Shih-Syong Dai, Hung-Chun Liao, Kit Man Chai, Yu-Siang Su, Tsung-Hsien Chuang, Shih-Jen Liu, Hsin-Wei Chen, Horng-Yunn Dou, Feng-Jui Chen, Chiung-Tong Chen, Chin-Len Liao, Guann-Yi Yu

**Affiliations:** ^1^National Institute of Infectious Diseases and Vaccinology, National Health Research Institutes, Zhunan, Taiwan; ^2^Immunology Research Center, National Health Research Institutes, Zhunan, Taiwan; ^3^Department of Biological Science and Technology, National Yang Ming Chiao Tung University, Hsinchu, Taiwan; ^4^Graduate Institute of Biomedical Sciences, China Medical University, Taichung, Taiwan; ^5^Graduate Institute of Medicine, College of Medicine, Kaohsiung Medical University, Kaohsiung, Taiwan; ^6^Institute of Biotechnology and Pharmaceutical Research, National Health Research Institutes, Zhunan, Taiwan

**Keywords:** SARS-CoV-2 Spike, VSV, pseudotype, replication-competent, S1/S2 cleavage site, furin, TMPRSS2, ACE2 transgenic mice

## Abstract

An effective COVID-19 vaccine against broad SARS-CoV-2 variants is still an unmet need. In the study, the vesicular stomatitis virus (VSV)-based vector was used to express the SARS-CoV-2 Spike protein to identify better vaccine designs. The replication-competent of the recombinant VSV-spike virus with C-terminal 19 amino acid truncation (SΔ19 Rep) was generated. A single dose of SΔ19 Rep intranasal vaccination is sufficient to induce protective immunity against SARS-CoV-2 infection in hamsters. All the clones isolated from the SΔ19 Rep virus contained R682G mutation located at the Furin cleavage site. An additional S813Y mutation close to the TMPRSS2 cleavage site was identified in some clones. The enzymatic processing of S protein was blocked by these mutations. The vaccination of the R682G-S813Y virus produced a high antibody response against S protein and a robust S protein-specific CD8^+^ T cell response. The vaccinated animals were protected from the lethal SARS-CoV-2 (delta variant) challenge. The S antigen with resistance to enzymatic processes by Furin and TMPRSS2 will provide better immunogenicity for vaccine design.

## Introduction

A novel coronavirus, severe acute respiratory syndrome coronavirus 2 (SARS-CoV-2), emerged in Wuhan, China in December 2019 and caused a coronavirus disease (COVID-19) pandemic. SARS-CoV-2 belongs to genus *Coronavirus* and family *Coronaviridae*. Typical symptoms of COVID-19 patients are fever or chills, dry cough, body aches, diarrhea, and loss of smell and taste (1). In severe cases, pneumonia and death can also occur. With the continuous circulation globally, SARS-CoV-2 variants have emerged from various countries and become dominant strain from time to time. Variants of concern (VOC) are variants that have high transmissibility, cause severe diseases, and reduce the effectiveness of vaccines or the accuracy of diagnostic detection. B.1.1.7 (Alpha), B.1.351 (Beta), P.1 (Gamma), B.1.617.2 (Delta), and B.1.1.529 (Omicron) are examples of VOC in the past two years. New generations of vaccines and therapeutics are still in urgent need.

The spike (S) protein is located on the viral envelope and interacts with the cellular receptor, Angiotensin-converting enzyme 2 (ACE2), during virus infection (2). S glycoproteins form homotrimers and are processed into S1 and S2 subunits by Furin protease (3, 4). The receptor-binding domain (RBD) of the S1 subunit interacts with ACE2 during infection, and the S2 subunit is subsequently cleaved by TMPRSS2 protease to trigger virus-host membrane fusion (5). The enzymatic processes are critical to enhancing SARS-CoV-2 propagation in lung (6, 7). The conformation of S protein changes from closed to open state to expose the RBD region in the prefusion states for ACE2 binding. Potent neutralizing antibodies isolated from the recovered COVID-19 patients have been mapped to the N-terminal domain and RBD region on open or closed conformation (8). Current COVID-19 vaccine designs use S protein as the target antigen for the induction of neutralizing antibodies to block SARS-CoV-2 entry (9, 10). Two proline substitution (2P), furin site mutation, and intermolecular disulfide bonds have been applied in the expression of the thermostable, closed S trimer as immunogen (3, 11–13).

The vesicular stomatitis virus (VSV) is a member of the *Rhabdoviridae* family and its genome only encodes five proteins: nucleoprotein (N), phosphoprotein (P), matrix protein (M), glycoprotein (G), and large polymerase protein (L). VSV G protein is responsible for viral binding, cell fusion, endocytosis, and entry. The VSVΔG vector has been applied to create recombinant viruses carrying the viral envelope protein (glycoprotein) from high pathogenic viruses for neutralization activity, antiviral evaluation, or vaccine design (14–16). The VSVΔG vector also has been used to express SARS-CoV-2 S protein for vaccine development (17, 18).

In this study, the replication-competent VSV-based SARS-CoV-2 S vaccine was generated, and the recombinant virus clones with different mutations were further characterized. The mutant containing mutations on the Furin and TMPRSS2 cleavage sites had higher potency to induce both humoral and cellular immunity against lethal SARS-CoV-2 infection. The information will advance our knowledge in vaccine design against emerging infectious diseases.

## Results

### Generation of Replication-Competent Recombinant VSV Expressing SARS-CoV-2 S Protein

**Figure 1:**
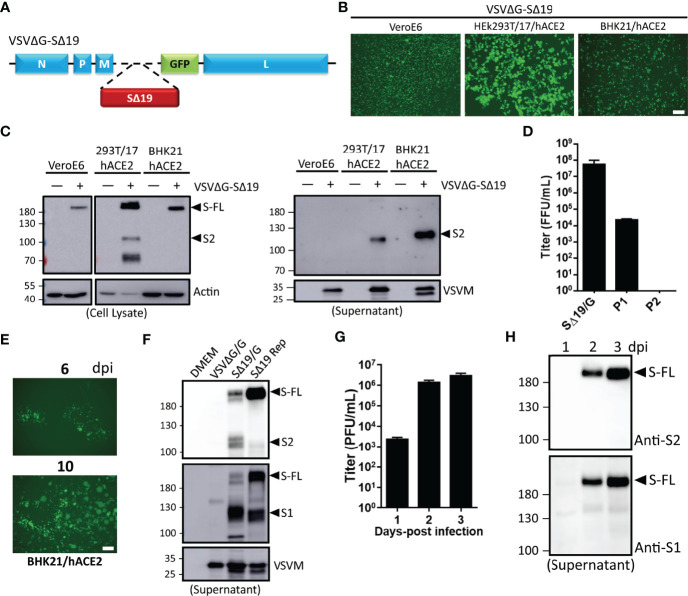
Generation of recombinant vesicular stomatitis virus (VSV) expressing the C-terminal 19 amino acid deletion SARS-CoV-2 spike mutant (VSVΔG-SΔ19) with replication capability. **(A)** Schematic representation of the genomic organization of the G protein-deficient VSV vector (VSVΔG) inserted with the C-terminal 19 amino acid truncated SARS-CoV-2 spike protein (SΔ19). N, nucleoprotein; P, phosphoprotein; M, matrix; GFP, green fluorescent protein; L, large polymerase. **(B, C)** Vero E6, HEK293T-hACE2, and BHK21-hACE2 cells were infected with the VSVΔG-SΔ19 virus (early passage, moi=0.5). Virus-driven GFP expression was monitored by fluorescence microscopy at 24 hrs post-infection **(B)**. The SΔ19 protein expression in cells (left panel) and supernatant (right panel) was examined by immunoblotting **(C)**. **(D)** The VSVΔG-SΔ19 virus was propagated in HEK293T-hACE2 cells, and the virus titer was determined in BHK21-hACE2 cells. **(E)** After a few passages in HEK293T-hACE2 cells, the viruses were inoculated in BHK21-hACE2 cells with limiting dilution. The replication-competent VSVΔG-SΔ19 viruses (SΔ19 Rep) emerging from BHK21-hACE2 cells were observed with GFP monitoring. **(F)** The S protein expression on recombinant virus particles of VSVΔG/G, the early passage of VSVΔG-SΔ19 enveloped with the VSV glycoprotein (SΔ19/G; replication incompetent) and SΔ19 Rep virus was examined by immunoblotting. **(G, H)** Vero E6 cells were infected with the SΔ19 Rep virus (moi=1, n=3). Virus titer **(G)** and S protein expression on virus particles **(H)** in culture supernatant were examined. moi, multiplicity of infection; dpi, days post-infection. Scale bar: 100 µm.

VSVΔG vector to express glycoprotein from high pathogenic virus has been applied in vaccine development. Hence, the human codon-optimized Spike cDNA lacking C-terminal ER retention signal (SΔ19) was inserted into the VSVΔG-GFP DNA vector for *in vivo* gene expression as depicted in **Figure 1A**. The VSVΔG-SΔ19 RNA genome was then rescued with helper plasmids (VSV N, P, L, and G) in the presence of T7 polymerase expression (19). The rescued virus particles enveloped with VSV G protein (VSVΔG-SΔ19/G) were infectious to drive GFP and S protein expression in Vero E6, HEK293T-hACE2, and BHK21-hACE2 cells (**Figures 1B, C**). It has been shown that ACE2 is the main receptor for SARS-CoV-2 infection, and human ACE2 expression in BHK-21 and A549 cells enhances the VSV-based S pseudotyped virus and rVSV-S infection (20, 21). Therefore, the hACE2-overexpressing HEK293T and BHK-21 were used for rVSV-S infection and related experiments. A substantial amount of VSVΔG-SΔ19 virus secretion in the culture supernatant was detected from HEK293T-hACE2 and BHK21-hACE2 cells (**Figure 1C**), but the virus titer decreased during passages (**Figure 1D**).

Recent studies showed that VSVΔG-SARS-CoV-2 S has the capability to generate replication-competent viruses with few mutations in the S gene (20, 22). After several repeated passages in BHK21-hACE2 cells, the virus-infected cell clusters (GFP^+^) emerged during the limiting dilution condition (**Figure 1E**), and the emerged replication-competent virus (SΔ19 Rep) could be further expanded in HEK293T-hACE2 cells at high titer. To further confirm the incorporation of S protein into SΔ19 Rep viral particles, immunoblotting was performed (**Figure 1F**). The SΔ19 Rep virus contained more uncleaved S protein compared to the non-replicating virus, VSVΔG-SΔ19/G, in which the viral particles were enveloped with VSV G protein to maintain virus production. Moreover, the SΔ19 Rep virus was able to propagate efficiently in Vero E6 cells with high yield (**Figure 1G**), and the SΔ19 protein on virus particles was predominantly as the uncleaved form (**Figure 1H**). In summary, replication-competent SΔ19 Rep could be generated and propagated efficiently in cells.

### Intranasal Vaccination of SΔ19 Rep Virus Effectively Induces Anti-spike Antibodies With Neutralizing Activity in Hamsters

**Figure 2:**
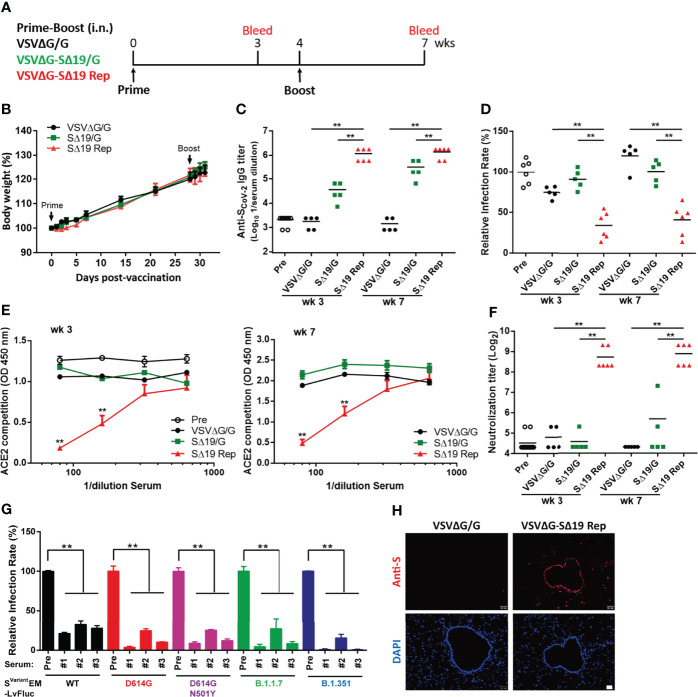
The SΔ19 Rep virus effectively stimulates anti-spike antibodies with neutralizing activity in hamsters. **(A)** Experimental design of the prime-boost vaccination with VSVΔG/G (control virus), VSVΔG-SΔ19/G (replication-incompetent virus), and SΔ19 Rep in Gold Syrian hamster by intranasal (i.n.) administration (1×10^6^ pfu or ffu/hamster; n=5-6). Body weight of vaccinated hamsters was monitored periodically **(B)**. Serum samples collected from 3 and 7 weeks post-vaccination were subjected to ELISA for anti-spike specific IgG antibody **(C)**, the VSVΔG-Spike pseudovirus-based neutralization assay **(D)**, the RBD-hACE2 interaction competition assay **(E)**, the SARS-CoV-2 neutralization assay **(F)**, and the the S^variant^ EM-LvFluc viruses-based neutralization assay **(G)**. **(H)** Lung sections obtained from hamsters infected with VSVΔG/G or SΔ19 Rep (1×10^6^ pfu or ffu/hamster; i.n.) at 20 hrs post-infection were stained with anti-S antibodies and counterstained with DAPI. **P < 0.01. Scale bar: 20 μm.

Recent studies have demonstrated that VSV expressing the S protein can be a vaccine candidate against SARS-CoV-2 (17, 18, 23). To examine whether VSVΔG-SΔ19 virus with replication activity could stimulate better protective immunity against SARS-CoV-2 infection, Golden Syrian hamsters (24, 25) were immunized twice with VSVΔG/G (vector control), SΔ19/G, or SΔ19 Rep virus *via* intranasal administration (i.n.; 10^6^ ffu or pfu/hamster) as depicted in **Figure 2A**. The immunized-hamsters did not show significant weight loss after virus administration, suggesting that the immunization was well tolerated (**Figure 2B**).

To evaluate the immune response, anti-S antibody titers in serum collected from Week 3 and 7 were evaluated by ELISA (**Figure 2C**). The SΔ19 Rep virus induced high anti-S antibodies in the hamsters on Week 3, and the antibody titer remained high at Week 7. In contrast, the anti-S antibody titer was low in the SΔ19/G-immunized hamsters on Week 3 and slightly elevated after the boost immunization on Week 7. The VSVΔG/G-immunized hamster did not have anti-S antibodies in the blood. Interestingly, both the Week 3 and 7 antiserum from the SΔ19 Rep-immunized hamsters could block VSVΔG-Spike pseudovirus infection (**Figure 2D**), interfere with RBD-hACE2 interaction (**Figure 2E**), and neutralize SARS-CoV-2 virus (hCoV-19/Taiwan/4/2020) infection in Vero E6 cells (**Figure 2F**). On the contrary, anti-S antibodies in the SΔ19/G-immunized hamsters did not show much neutralizing activity in these assays. In addition, the lentiviral vector with firefly luciferase reporter packaged with E, M, and S variants (S^variant^ EM-LvFluc), including S^D614G^, S^D614G+N501Y^, S^B1.1.7^, and S^B1.351^, were generated and used for neutralization tests (26) (**Figure 2G**). The SΔ19 Rep antisera from Week 7 effectively blocked S^WT^ and S^variant^ pseudovirus infection. To examine whether S protein expression was stimulated by the SΔ19 Rep virus vaccination in hamsters, the lung tissues were collected at 20 hrs post-vaccination and subjected to immunofluorescent staining with anti-S antibody (**Figure 2H**). The S protein expression was induced by the SΔ19 Rep virus in the epithelial cells in the trachea and scattered in a few surrounding pneumocytes, suggesting that the SΔ19 Rep undergoes transient replication in the lung to stimulate immune responses. Collectively, these data show that the intranasal administration of the replication-competent SΔ19 Rep virus efficiently induces anti-S antibodies with potent and broad neutralizing activity against SARS-CoV-2.

### Prime-Boost or a Single Dose of the SΔ19 Rep Vaccination Protects Hamsters From SARS-CoV-2 Infection

**Figure 3:**
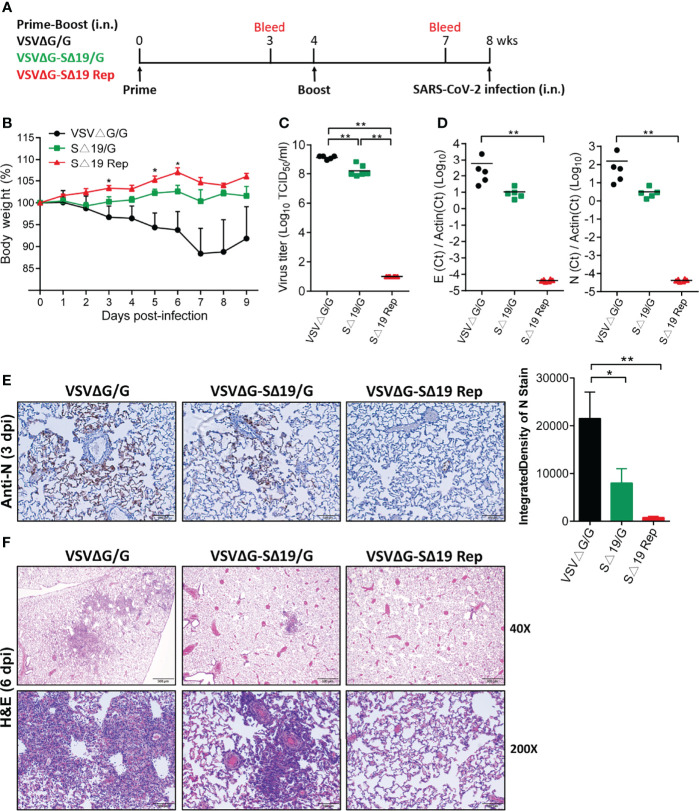
Prime-boost SΔ19 Rep vaccination protects hamsters from SARS-CoV-2 infection. **(A)** Experimental design of the prime-boost vaccination and SARS-CoV-2 challenge (1×10^5^ TCID_50_/hamster; i.n.) in hamsters. **(B)** Body weight loss post-SARS-CoV-2 challenge. **(C)** The virus titers of SARS-CoV-2 in the lung at 3 dpi were detected by TCID_50_ assay. **(D)** Viral RNA transcripts of E and N genes in the lung (3 dpi) were measured by quantitative real-time PCR. **(E)** Viral N protein expression in the lung (3 dpi) was detected by immunohistochemistry and quantified using ImageJ software. Scale bar: 100 μm. **(F)** H&E staining of lung tissue at 6 dpi. Scale bar: 500 μm (40X) and 100 μm (200X). *P < 0.05; **P < 0.01.

To evaluate the efficacy of the SΔ19 Rep vaccination, the prime-boost immunized hamsters were challenged with SARS-CoV-2 (1x10^5^ TCID_50_/hamster; i.n.; **Figure 3A**). The VSVΔG/G-immunized hamsters showed body weight loss and gradually recovered after day 7-post infection (dpi) (**Figure 3B**). The SΔ19/G-immunized hamsters had slight body weight loss only in the first few days after infection. In contrast, the SΔ19 Rep-immunized animals did not have obvious weight loss throughout the experiment. The virus titer in lungs from the VSVΔG-immunized hamsters was up to 1x10^9^ TCID_50_/ml (1/2 lung was homogenized in 2ml) on 3 dpi, and it was decreased to one-tenth in the lungs from the SΔ19/G-immunized animals (**Figure 3C**). Surprisingly, the SARS-CoV-2 virus was not detected in the lungs obtained from the SΔ19 Rep-immunized animals. The viral E and N RNA were also not detected in the 3 dpi lungs from the SΔ19 Rep-immunized animals evaluated by quantitative real-time PCR (**Figure 3D**). Consistently, the SARS-CoV-2 N antigen was highly expressed in the VSVΔG/G-immunized lungs by immunohistochemistry staining, decreased in the SΔ19/G-immunized lungs, and absent in the SΔ19 Rep-immunized lungs (3 dpi, **Figure 3E**). The pulmonary inflammation caused by SARS-CoV-2 infection was evident in the VSVΔG/G-immunized lungs at 6 dpi with hematoxylin & eosin staining but not observed in the SΔ19 Rep-immunized lungs (**Figure 3F**).

**Figure 4:**
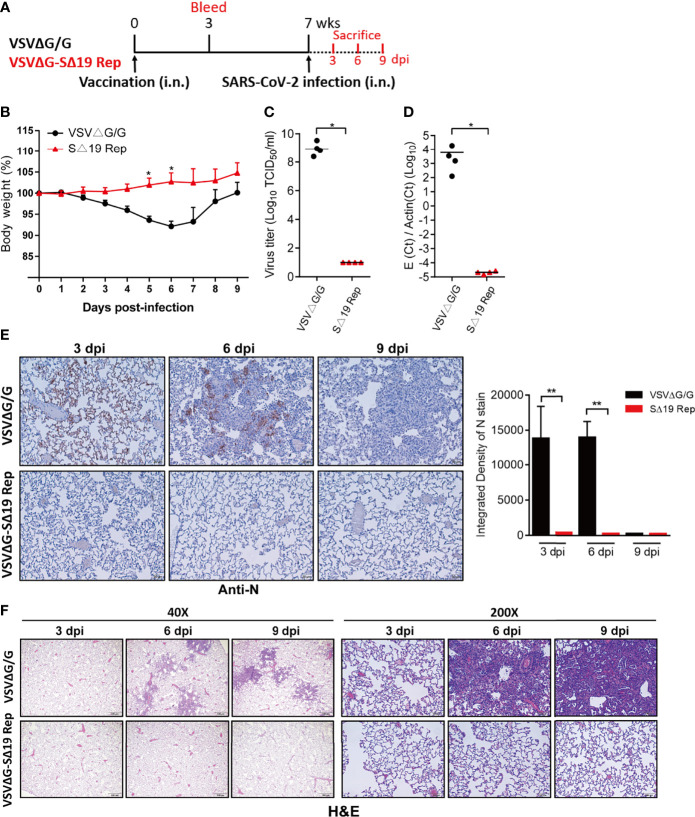
A single dose SΔ19 Rep vaccination induces effective immunity against SARS-CoV-2 infection. **(A)** Experimental design of a single dose vaccination (1×10^6^ pfu or ffu/hamster; i.n.; n=10) and SARS-CoV-2 challenge (1×10^5^ TCID_50_/hamster; i.n.). **(B)** Body weight loss post-SARS-CoV-2 challenge. SARS-CoV-2 virus titer **(C)**, viral RNA **(D)**, and N protein expression **(E)** in the lung at 3 dpi were assessed. Scale bar: 20 μm. **(F)** H&E staining of lung tissue (3, 6, and 9 dpi). Scale bar: 500 μm (40X) and 100 μm (200X). *P < 0.05; **P < 0.01.

As a single dose of the SΔ19 Rep vaccination could induce high titer anti-S antibodies with neutralization activity in hamsters (Week 3 in **Figures 2C**–**F**), a single dose of the SΔ19 Rep vaccination might be protective to SARS-CoV-2 infection. Therefore, hamsters with one dose of SΔ19 Rep immunization were challenged with SARS-CoV-2 infection (1x10^5^ TCID_50_/hamster; i.n.; **Figure 4A**). The hamsters with one dose of VSVΔG/G immunization were used as a control group. Similarly, the SΔ19 Rep-immunized hamsters were resistant to SARS-CoV-2 infection and therefore did not show body weight loss after infection (**Figure 4B**). No virus titer, viral RNA, and N protein expression were observed in the lung with the SΔ19 Rep vaccination at 3 dpi (**Figures 4C**–**E**). The inflammation induced by the SARS-CoV-2 infection was also absent in the SΔ19 Rep-immunized lung tissue at 6 dpi (**Figure 4F**). Taken together, a single dose of SΔ19 Rep intranasal vaccination is sufficient to induce robust immunity against SARS-CoV-2 infection.

### R682G and S813Y Mutations Reduce the Substrate-Protease Binding Affinity

**Figure 5:**
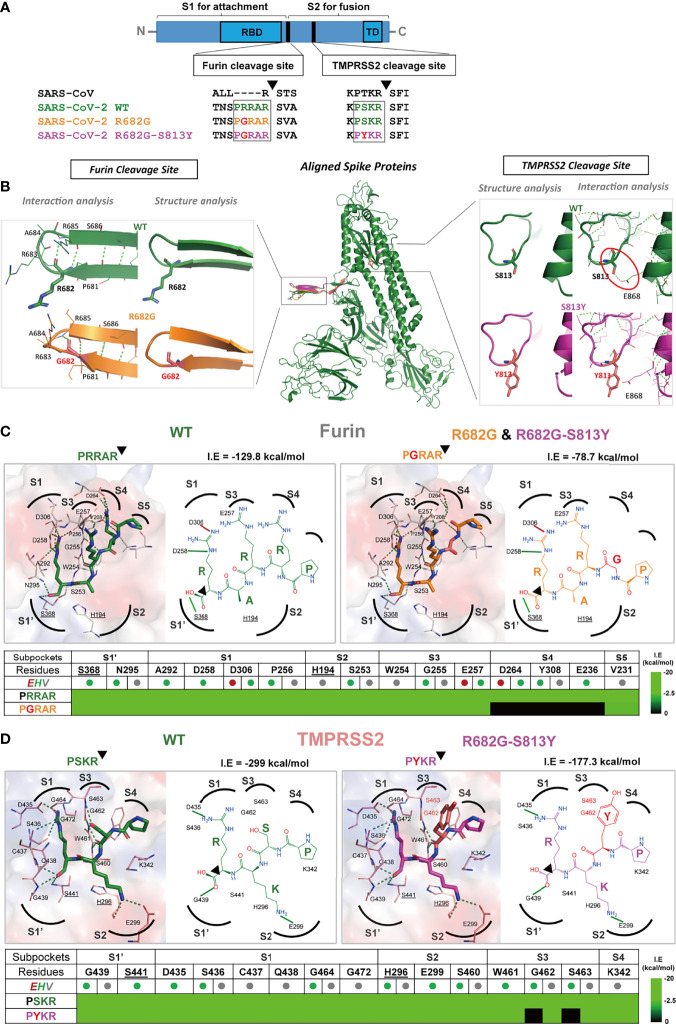
The R682G and S813Y mutations in Spike protein lead to altered enzyme binding and proteolysis. **(A)** The Furin and TMPRSS2 cleavage motifs are shown in the spike protein sequences of SARS-CoV, SARS-CoV-2 WT, and mutants. Furin cleavage occurs between the S1 and S2 subunits, while the TMPRSS2 cleavage occurs within the S2 subunit. **(B)** The S protein structure and interactions of the WT (wild-type; green), R682G (orange), and R682G-S813Y (magenta) mutants were modeled using the Phyre2 web portal. The full spike proteins are aligned, and the specific mutation sites are shown in insights of the Furin and TMPRSS2 cleavage sites. **(C, D)** Molecular docking of spike protein peptide substrates (of WT and mutants) with Furin and TMPRSS2 using iGEMDOCK. The protease active site (surface) and the binding poses (2D diagrams) of the WT (green stick) and mutants (mutated residue in red sticks) were shown. The active site subpockets (dotted curves) with residues (catalytic residues labels underlined) were also displayed. The interactions (solid black lines, E-dark red, H-green, V-grey) and total interaction energies (I.E in kcal/mol) were examined. The interaction table details the substrate-subpocket residue interactions.

Recent studies showed that the replication-competent VSVΔG-SARS-CoV-2 S harbors unique adoptive mutations (18, 20, 22, 27). To examine whether any mutations had accumulated in the SΔ19 Rep virus, the viral RNAs extracted from cell culture supernatant were subjected to amplicon sequencing assay utilizing the ONT MinION device (28). The mutations of the dominant strain that occurred in the viral protein-coding sequence were listed in **Table S1**. Two nonsynonymous substitutions, R682G (74.10%) and S813Y (46.22%) were identified in the SΔ19 gene, which are close to the Furin- and TMPRSS2-cleavage sites, respectively (**Figure 5A**). To further characterize the R682G and S813Y mutations on SΔ19 Rep virus, viral clones were isolated *via* limiting dilution in BHK21-hACE2 cells and then amplified in HEK293T-hACE2 cells (**Figure S1A**). Some viral clones lost GFP expression but caused obvious CPE. 9 viral clones with the stable S protein expression were subjected sequencing (**Figures S1B, C**). All the clones sequenced had R682G mutation, and all the GFP^-^ clones sequenced had S813Y mutation. Two additional mutations, P793H and R1107H, were also identified in some clones.

The R682G mutation in the S protein slightly changed the conformation of the loop (**Figure 5B**) but did not cause any change in the interactions with the neighboring residues, thus the spike structural flexibility and stability are unaffected by this mutation. The mutation of S813 to Y813 in the loop (near the TMPRSS2 cleavage site) does not affect the local loop structure. However, the H-bonding interaction of S813 with E868 residue of the adjacent helix is lost in the mutant that contains Y813 (red circle in **Figure 5B**) and may alter the S protein structural stability.

Furin and TMPRSS2 are important human proteases essential for SARS-CoV-2 S protein cleavage and maturation, leading to RBD opening and binding to host ACE2 receptor to mediate virus entry (29). To further examine the effect of mutations in the protease substrate motifs on proteolysis, their binding mechanisms with proteases were evaluated by iGEMDOCK (30) (**Figures 5C, D**). In Furin protease (**Figure 5B**), the peptide substrates ^681^P**R**RAR^685^ (WT) and ^681^P**G**RAR^685^ (R682G mutant) were docked into the active site and interacted with the catalytic residues S368 and H194. At the S4 subpocket, R682 residue interacts with subpocket residues D264, Y308, and E236, contributing to strong substrate binding with an I.E of -129.8 kcal/mol. However, the R682G mutation disrupted these interactions leading to an unoccupied S4 subpocket (Table in **Figure 5C**) and thus an overall weakened binding of the peptide with I.E of -78.7 kcal/mol.

The binding poses of ^812^P**S**KR^815^ (WT) and ^812^P**Y**KR^815^ (S813Y mutant) in the TMPRSS2 active site subpockets were also analyzed (**Figure 5D**). The PSKR engaged S1’ and S1-S4 subpockets by interaction with residues including catalytic S441 and H296, where Ser (S) strongly forms H-bonding with the S3 subpocket residues G462 and S463 (red outline in **Figure 5D**), with an overall I.E of -299 kcal/mol. These H-bonding interactions at S3 are missing in the PYKR substrate-binding pose due to mutation to Tyr (Y), also observed in the interaction table in **Figure 5D**. This mutation decreases the overall interaction energy I.E to -177.3 kcal/mol, and it reduces binding affinity for mutant substrate peptides and the rate of proteolysis by TMPRSS2 compared to WT. These docking results indicated that the R682G and S813Y mutations reduced the substrate-protease binding affinity to Furin and TMPRSS2, respectively, and thus may hinder the proteolysis process.

### The R682G and S813Y Mutations of S Protein Facilitate Recombinant Virus Production

**Figure 6:**
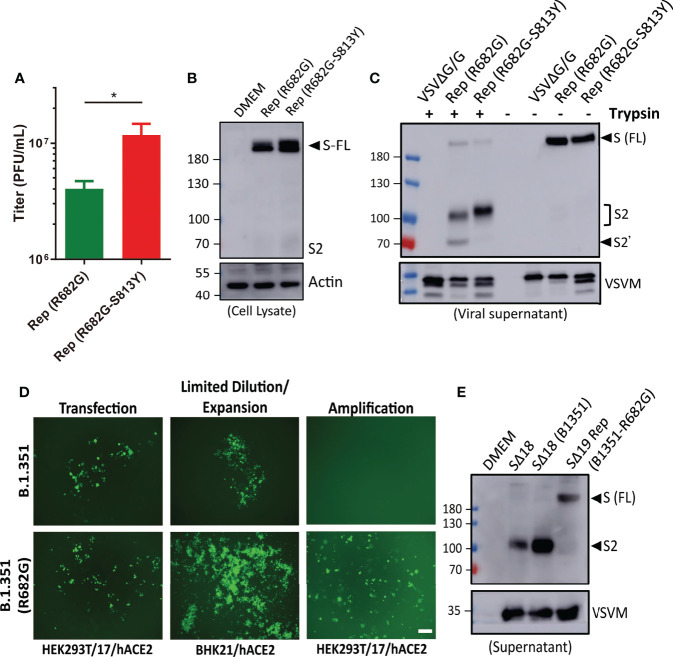
R682G and S813Y mutations facilitate rVSV-S replication. **(A)** HEK293T-hACE2 cells were infected with the SΔ19 Rep virus with R682G or R682G-S813Y mutation. Virus titer in the culture supernatant was measured (moi=0.1, 3 dpi, n=4). **(B)** Virus-infected cell lysate (moi=1, 1 dpi) was subjected to immunoblotting with anti-S2 antibodies. **(C)** The viral particles in the culture supernatant were digested with TPCK-trypsin (2 μg/ml at 37°C for 30 min) and analyzed by immunoblotting. **(D)** SΔ19 beta variant (B.1.351) gene with and without R682G mutation was inserted into the VSVΔG-GFP DNA vector and co-transfected with all the required helper plasmids in HEK293T-hACE2 cells to rescue recombinant VSVΔG-SΔ19 (B.1.351)/G virus. Virus replication was monitored by GFP expression. The rescued viruses were collected to infect BHK21-hACE2 cells with limiting dilution, and further amplified in HEK293T-hACE2 cells. **(E)** The Spike protein expression pattern of the replication-competent SΔ19 (B.1.351-R682G) virus was examined by immunoblotting alone with SΔ18 and SΔ18 (B.1.351) pseudoviruses for comparison. Scale bar: 100 μm. *P < 0.05.

When the SΔ19 Rep clones with R682G and R682G-S813Y mutations were amplified in HEK293T-hACE2 cells, the virus production was higher in the R682G-S813Y mutant (**Figure 6A**). The S protein expression in the R682G and R682G-S813Y mutant virus-infected cells was shown as a full-length form which was due to the inhibition of the S1/S2 cleavage by R682G mutation (**Figure 6B**). To test whether S813Y mutation changes S protein structure and protease sensitivity, the SΔ19 Rep viruses were treated with TPCK-trypsin. As shown in **Figure 6C**, the S2 protein and a smaller fragment (S2’) appeared in the R682G mutant after trypsin digestion. In contrast, the S2’ fragment was not generated from the R682G-S813Y mutant virus with trypsin digestion, suggesting that the S813Y mutation changes the S protein to become resistant to enzymatic processing.

As all the SΔ19 Rep clones harbored R682G mutation, we suspect that the mutation might stabilize S protein as full-length form and accelerate recombinant VSVΔG-SΔ19 virus to become replication-competent. To test the hypothesis, recombinant VSVΔG-SΔ19 (B.1.351) -WT or -R682G mutant constructs were made, and virus production was monitored by GFP expression (**Figure 6D**). The replication-competent virus of R682G mutant was emerged after being expended in the BHK21-hACE2 cells, which did not occur in the WT group. The S protein in the SΔ19 (B1351-R682G) Rep virus was also present as an uncleaved form (**Figure 6E**), and no additional adaptive mutation was identified in the S gene of the viral RNA. Taken together, the R682G mutation blocks the S1/S2 cleavage and facilitates Rep virus packaging. The S813Y mutation attenuates further processing of S2 protein.

### The SΔ19 Rep R682G-S813Y Mutant Stimulates Robust Antibody Response and Th1 Response

**Figure 7:**
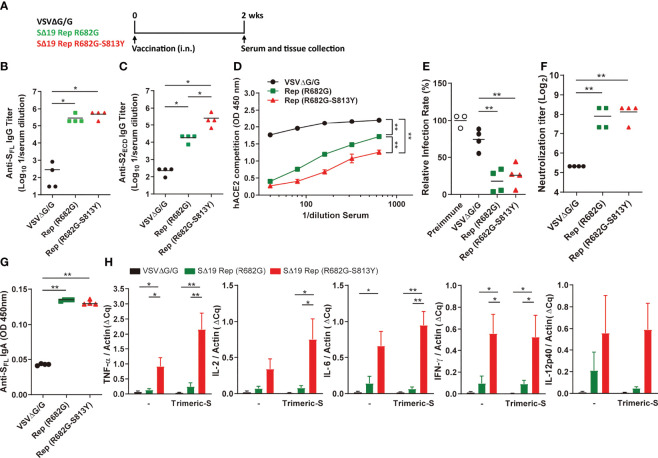
The SΔ19 Rep (R682G-S813Y) mutant stimulates robust neutralizing antibodies and Th1 immune response in hamsters. **(A)** Experimental design of a single dose vaccination with VSVΔG/G (control virus), SΔ19 Rep-R682G and SΔ19 Rep-R682G-S813Y in hamster (i.n.; 1×10^6^ pfu or ffu/hamster; n=4). The serum, spleen, and lung of the vaccinated hamsters were collected 2 weeks post-vaccination. Serum samples were subjected to anti-S_FL_ **(B)** and anti-S2_ECD_ **(C)** IgG ELISA, the RBD-hACE2 interaction competition assay **(D)**, and the neutralization assay with the VSVΔG-based S pseudotyped virus **(E)** and SARS-CoV-2 virus **(F)**. The anti-S_FL_ IgA expression in the lung homogenates was measured by ELISA **(G)**. The isolated hamster splenocytes were stimulated with or without trimeric-S protein (5 μg/ml) for 72 hrs, and subjected to detect the TNF-α, IL-2, IL-6, IFN-γ and IL-12 mRNA expression by qRT-PCR **(H)**. *P < 0.05; **P < 0.01.

It is known that SARS-CoV-2 S with Furin cleavage site mutation and S-2P substitutions is stabilized in the prefusion state to stimulate better immune responses (11, 31). The R682G and R682G-S813Y mutants have lower sensitivity to enzymatic processes (14, 32), which might enhance their potency during vaccination. To test the hypothesis, hamsters were immunized with R682G or R682G-S813Y mutants, and their immune responses were analyzed at two weeks post-immunization (**Figure 7A**). All the immunized-hamsters did not show significant weight loss after immunization (**Figure S2A**). Both R682G and R682G-S813Y mutants induced a high titer of antibodies against full-length S protein in the serum (**Figure 7B**). Interestingly, the R682G-S813Y mutant stimulated a significantly higher titer of antibodies against the S2 region compared to the R682G mutant and the VSVΔG/G virus (**Figure 7C**). In addition, the serum collected from the R682G-S813Y mutant-immunized animals blocked the RBD-hACE2 interaction more effectively (**Figure 7D**). The serum collected from the R682G and R682G-S813Y groups could neutralize the VSV-based S pseudoviruses (**Figure 7E** and **Figure S2B**) and SARS-CoV-2 virus (**Figure 7F**), but not much difference was observed between these two groups. IgA antibodies against the S protein were also elevated in lung homogenates from the R682G and R682G-S813Y groups (**Figure 7G**). Taken together, intranasal vaccination of these two SΔ19 Rep strains stimulate a robust humoral immune response in hamster.

Type 1 T helper (Th1) cells are important in the activation of the cellular immune responses, which are critical for virus infection control. Whether the SΔ19 Rep vaccination could stimulate cellular immunity was further evaluated. The spleen tissue was collected from the vaccinated-hamsters and subjected to splenocyte culture with or without trimeric-S protein stimulation for three days. When the cytokine expression was analyzed by qRT-PCR, the Th1-related cytokines, including TNF-α, IL-2, IL-6, IFN-γ, and IL-12, were highly elevated in the splenocytes isolated from the SΔ19 Rep R682G-S813Y-vaccinated hamsters (**Figure 7H**). The Th2 related cytokine expression (IL-4, IL-5, and IL-13) in the isolated splenocytes was not detected. The expression of TNF-α, IL-2, IL-6, IFN-γ, and IL-12 mRNA were also elevated in the spleen tissue but no significant difference between the R682G and R682G-S813Y groups (**Figure S2C**). These results suggest that the SΔ19 Rep R682G-S813Y virus could simultaneously induce humoral and cellular immunity against SRAS-CoV-2 S protein.

### Vaccination With the R682G-S813Y Mutant Protects Mice From the SARS-CoV-2 Lethal Infection

**Figure 8:**
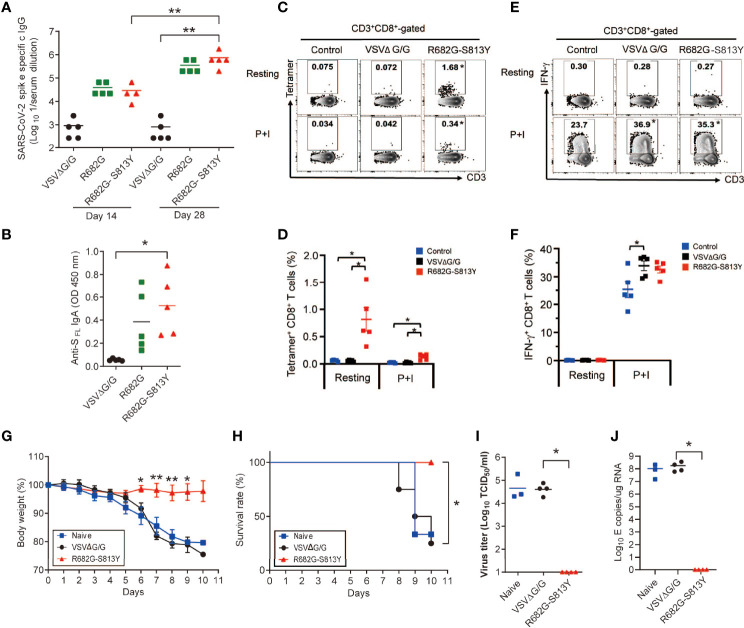
The SΔ19 Rep (R682G-S813Y)-vaccinated hACE2 transgenic mice were protected from the SARS-CoV-2 lethal infection. **(A)** K18-hACE2 transgenic mice were vaccinated with VSVΔG/G (control virus), SΔ19 Rep-R682G, and SΔ19 Rep-R682G-S813Y twice on Day 0 and Day 21 (i.n.; 1×10^8^ pfu or ffu/mouse; n=5). Anti-S_FL_ IgG titer in the serum samples collected on Day 14 and Day 28 was measured by ELISA. **(B)** Anti-S_FL_ IgA titer in Nasal lavage fluid (Day 35) was detected by ELISA. **(C–F)** Splenocytes were isolated on Day 35-post vaccination and stained with T cell markers and the H-2K(b) SARS-CoV-2 S Tetramer for flow cytometry. Some splenocytes were treated with PMA (50 ng/ml) and ionomycin (500 ng/ml) for 5 h to activate T cell (P+I). **(G–J)** The vaccinated-mice were challenged with the SARS-CoV-2 Delta strain (1000 TCID_50_/mouse; i.n.; n=6-8). Mouse body weight loss **(G)** and survival rate **(H)** were monitored. Half of the infected-mice were sacrificed, and the virus titer **(I)** and viral RNA level **(J)** in the mouse lung were measured. *P < 0.05; **P < 0.01.

To examine the SΔ19 Rep infectivity and the duration of S protein expression in mice, the K18-hACE2 transgenic mice were infected with the SΔ19 Rep R682G-S813Y virus (1x10^8^ pfu/mouse; i.n.), and the expression of S protein in lung collected from Day 1 to 4 post-infection was monitored by immunostaining (**Figure S3**). The transient S protein expression was detected only in the lung sections on Day1 post-infection. To evaluate whether the SΔ19 Rep vaccination could induce protective immunity against SARS-CoV-2 lethal infection, K18-hACE2 transgenic mice were immunized with the R682G and R682G-S813Y viruses. As mice seemed to have a strong innate immune response against the VSV vector, a high dose of SΔ19 Rep virus (1x10^8^ pfu/mouse) and the prime-boost protocol were used to vaccinate the hACE2 mice. The S protein-specific antibodies and CD8^+^ T cell responses were evaluated and compared between those immunizations. On day 14 post the prime-boost, the vaccination with R682G viruses as well as R682G-S813Y viruses led to a 10 to 20-fold induction of S protein-specific IgG in the serum compared to VSVΔG/G control (**Figure 8A**). The level of S protein-specific IgG induced by R682G-S813Y mutant increased persistently at day 28 after the prime-boost, suggesting a better S protein-specific antibody response of R682G-S813Y vaccine than R682G. Consistently, a significant induction of S protein-specific IgA was detected in the nasal lavage fluid of R682G-S813Y vaccinated-mice, which was less apparent in mice immunized with R682G virus (**Figure 8B**). We subsequently explored the induction of S protein-specific CD8^+^ T cells by the R682G-S813Y vaccine, which was required for a complete vaccine-mediated immune protection. To achieve this, splenocytes in the vaccinated-mice were harvested at day 35 post the prime-boost, left untreated (resting) or stimulated with PMA plus ionomycin (P+I) for 5 h in the 10% FBS/RPMI full medium, followed by the SARS-CoV-2 S protein-specific tetramer staining in CD8^+^ T cells. As expected, R682G-S813Y vaccine greatly induced S protein-specific tetramer^+^ CD8^+^ T cells, while VSVΔG/G control did not (**Figures 8C, D**). However, the stimulation with P+I did not induce more tetramer^+^ CD8^+^ T cells. Meanwhile, the R682G-S813Y vaccine induced the production of IFN-γ by CD8^+^ T cells significantly upon stimulation with P+I, which was similar to those by VSVΔG/G control (**Figures 8E, F**). The results suggested that most of the IFN-γ response in CD8^+^ T cells mediated by the R682G-S813Y vaccine was driven from the VSV vector. Taken together, the R682G-S813Y vaccine boosted a superior prime-induced immunity with higher levels of S protein-specific IgG and IgA, and a higher frequency of S protein-specific CD8^+^ T cells in mice.

When the K18-hACE2 mice were challenged with SARS-CoV-2 (Delta strain; 1000 TCID_50_/mouse), naïve or the VSVΔG/G vaccinated mice had obvious body weight loss and died on 8-10 dpi (**Figures 8G, H**). In contrast, the R682G-S813Y virus vaccinated mice did not show body weight loss and all mice survived on 10 dpi. Consistently, virus titer and viral RNA were not detected in the R682G-S813Y mouse group (**Figures 8I, J**), suggesting that these mice were fully protected from the virus challenge. In conclusion, antibody and T cell responses induced by the R682G-S813Y vaccine could protect the hACE2 transgenic mice from the lethal SARS-CoV-2 virus infection.

## Discussion

The recombinant VSV (rVSV)-vectored vaccine platform has been used for protection against several viral pathogens. For instance, the VSV-Ebola (rVSV-ZEBOV) was approved by the FDA in 2019. The VSV is not a human pathogen, so preexisting immunity against the VSV vector is not a concern. The replication-competent rVSV can induce effective humoral and cellular immunity. Safety-wise, rVSV replication only lasts for a short duration *in vivo* as VSV is very sensitive to *IFN*-mediated antiviral responses. In addition, the replication-competent rVSV is easy to propagate with a high titer in cell culture. These phenotypes prove the great advantage of the rVSV vaccines in future applications. Indeed, the SΔ19 Rep (R682G-S813Y) virus has high immunogenicity to induce high titer of IgG and IgA and robust T cell response. The vaccine strain will provide a new choice for COVID-19 vaccine development.

The Replication-competent VSV-vectored S vaccines have also been established previously (14, 18, 20, 23, 27). Mutations identified from the previous study were located in the Furin cleavage site or C-terminal tail-truncation. The C-terminal cytoplasmic tail of S protein contains an ER retention signal (KxHxx), which facilitates S protein processing at the ER-Golgi intermediate compartment (ERGIC) and virus particle assembly (4). As VSV assembly mainly occurs at the plasma membrane, removal of the ER retention signal could facilitate the rVSV-S assembly, infectivity, and enhance the expression of S protein (20, 27, 33). Mutation or deletion of the furin cleavage site of S protein reduces SARS-CoV-2 entry and cell-cell spread, which leads to reduced virulence and pathogenesis in hamsters (34). Cheng et al. showed that the R682A mutation blocks Furin cleavage and reduces virus infectivity (35). In contrast, mutations in the multi-basic motif of the Furin cleavage site in the S protein seem to provide a growth advantage for the replication-competent VSVΔG-S viruses (18, 20, 22). All the clones isolated in our study contain R682G mutation and the additional S813Y mutation located near the TMPRSS2 provide a growth advantage of the rVSV virus. Making the S protein resistant to the enzymatic processing is critical for the rVSV assembly and enhances virus production. The novel ribosomal pausing site, CGGCGG (RR) (36) was not present in our codon-optimized sequence (AGGAGA) or the R682G mutant (GGGAGA).

The conformation of S protein changes from closed to open state to expose the RBD region in the prefusion states for ACE2 binding. Furin cleavage at the S1/S2 junction promotes the conformation transition from closed to open state and facilitates virus infection. The R682G mutation reduces the furin cleavage efficiency of S protein, which may keep the S protein in a closed conformation. As a residual level of S1 and S2 are detected by immunoblotting, the proteolysis of the S1/S2 junction is not completely blocked by the R682G mutation. On the other hand, the slow processivity of the R682G and S813Y mutants might have a longer half-life for immune stimulation. Neutralizing antibodies mapped to the RBD domain on closed conformation have high potency to block SARS-CoV-2 infection (8). The conformation changes of the R682G and S813Y mutations may not affect the generation of neutralizing antibodies significantly. Johnson et al. showed that the SARS-CoV-2 virus with ΔPRRA mutation on S protein conferred protection against re-challenge with the parental SARS-CoV-2 (34). Probably due to the conformation changes or more intact S molecules on the ΔPRRA virions, the neutralization values of the sera from the COVID-19 patients were reduced against the ΔPRRA mutant versus parental virus. These phenotypes of ΔPRRA might also apply to the R682G and S813Y mutants.

Several studies showed that the furin cleavage site of S protein is required for SARS-CoV-2 virus entry and cell-cell fusion (7, 29, 37). Papa et al. showed that furin cleavage is not essential for processing S protein, and other proteases could cleave the S1/S2 junction (7). Current evidence suggests that SARS-CoV-2 enters cells through TMPRSS2- or cathepsin-meditated pathways (38, 39). The SARS-CoV-2 carrying with WT spike enters the cell preferentially *via* TMPRSS2-dependent pathway, whereas SARS-CoV-2 without the S1/S2 cleavage site infects cell *via* cathepsin-dependent pathway (40). With the reduced sensitivity to furin and TMPRSS2 cleavage, the rVSV-S R682G and R682G-S813Y viruses might enter cells through both TMPRSS2- or cathepsin-meditated pathways for infection and immune stimulation.

We suspect that the proteases-resistant S protein might be more stable and therefore serve as a better antigen for vaccination. Riley et al. resolved the structure of spike trimer by negative stain EM and cryo-EM (41) and showed that a higher percentage of the S protein without furin cleavage site remains as trimeric form compared to the wild-type S protein. Trimeric-S, which has the S1/S2 cleavage site replaced by GSAS, is a potent antigen for the induction of neutralization antibodies (3). In line with this, immunization of the recombinant S proteins carrying two proline substitution (2P) and furin site mutation completely protects mice from SARS-CoV-2 challenge and produces a higher serum neutralization titer compared to the wild type S antigen immunization in mice (42). The protease-resistant and 2P mutations to stabilized S antigens have been applied in several vaccine designs (31, 43). The SΔ19 Rep R682G virus induces a high neutralizing antibody response. The SΔ19 Rep R682G-S813Y virus stimulates better antibody response against S2 region and triggers a much stronger CD8^+^ T cell response. The S2 subunit is more conserved within coronaviruses and SRAS-CoV-2 variants. A neutralizing antibody specific to the S2 subunit with cross-reactivity to carious coronaviruses is recently identified (44). The R682G-S813Y mutation might sustain more extended rVSV replication in animals, and the mutant S antigen might also have a longer half-life for the immune cells to recognize. The mutations could be included in the new generation of vaccine design.

## Material and Methods

### Cells and Virus

BHK-21 cells were maintained in RPMI medium (Hyclone, Marlborough, MA) containing 5% FBS (Hyclone, Marlborough, MA), 1x Penicillin Streptomycin (PS) solution (Corning, New York). HEK293T/17 cells and Vero E6 cells were maintained in 10% FBS in DMEM/High glucose medium (Hyclone, Marlborough, MA) with 1X PS solution. Human ACE2 overexpression in BHK-21 and HEK293T cells was achieved following a previously published method (26). SARS-CoV-2 virus (hCoV-19/Taiwan/4/2020) and Delta virus (hCoV-19/Taiwan/1144/2021) were kindly provided by the Taiwan Centers for Disease Control and amplified in Vero76 cells in M199 medium with 2 μg/mL TPCK-trypsin. All SARS-CoV-2 experiments were performed in a biosafety level 3 (BSL-3) laboratory.

### VSVΔG/S Pseudovirus and the Replication-Competent VSVΔG-SΔ19 Virus

The VSVΔG-GFP/G virus was recovered from the VSVΔG-GFP-2.6 plasmid and helper plasmids (G, L, N, and P; Kerafast, Boston, MA) by following previously described methods (19). Human codon-optimized S cDNA encoding the WT (wild-type, MN908947.3) or variants were inserted in the pVax1 expression vector. Codon optimization is based on OptimumGene from GenScript. The BHK-21 cells were transfected with pVax1-SARS-CoV-2 S plasmid using lipofectamine 2000 (ThermoFisher, Waltham, MA) and infected with the VSVΔG-GFP/G virus (moi=5) the next day. After incubation for 24 hrs, the cultured supernatants were clarified by centrifugation at 1,320 × g for 10 min and stored at -80°C. For titration, BHK21-hACE2 cells were infected with serially-diluted S_pp_ stock, and the virus titer (ffu/ml) was calculated by counting GFP positive cells. The S cDNA lacking C-terminal ER retention signal (SΔ19) was PCR amplified and inserted into the VSV-ΔG-GFP-2.6 vector. The replication-competent SΔ19 virus was recovered by the following method described previously (22) and briefly described in the Supplementary Methods.

### Immunoblotting Analysis

Cells were lysed by lysis buffer (50 mM Tris, 250 mM NaCl, 3 mM EDTA, 1% Triton X-100, 0.5% NP-40, 10% Glycerol, 1x PI cocktail). Cell lysate (10-50 μg) or viral supernatant were subjected to SDS-PAGE and transferred onto PVDF membrane. The primary antibodies were used in the study: anti-SARS-CoV-2 S2 (Genetex, Hsinchu, Taiwan; Cat#632604), anti-VSV-M (Absolute, Boston, MA), and anti-beta-actin (Sigma, St. Louis, MO). The rabbit anti-RBD (RBD antigen purified from *E.coli*) was generated in-house. The secondary antibodies were purchased from Jackson immunoresearch (West Grove, PA). The detection signals were developed with SuperSignal West Pico PLUS Chemiluminescent Substrate (Thermo Scientific, Rockford, IL) and imaged by Amersham Imager 600 (Dealer GE Healthcare).

### Immunization and SARS-CoV-2 Challenging in Hamster and Mice

The 7-9 weeks old female Golden Syrian hamsters were purchased from the National Laboratory Animal Breeding and Research Center (Taipei, Taiwan). Hamsters were intranasally immunized with 1×10^6^ ffu or pfu of recombinant VSV viruses with a single dose or two doses at a 4 weeks interval. For SARS-CoV-2 challenge tests, immunized hamsters were intranasally inoculated with 1×10^5^ TCID_50_ of SARS-CoV-2 (hCoV-19/Taiwan/4/2020). K18-human ACE2 transgenic mice (32) were imported from the Jackson Laboratory and bred at BioLASCO Taiwan and NHRI. The transgenic mice were immunized with 1×10^8^ ffu or pfu of recombinant VSV viruses with boost doses at a 3 weeks interval.

### Enzyme-Linked Immunosorbent Assay

Serum, lung homogenate, and nasal lavage fluid (NLF) were subjected to ELISA to detect anti-S-specific IgG or IgA titer. 96-well ELISA plates were coated with either 4 μg/ml recombinant trimeric-S protein (S_FL_; homemade from 293T cells) or SARS-CoV-2 S2 extra-cellular domain (S2_ECD_, SinoBiologicals, Beijing, China) in PBS overnight at 4°C, and blocked with 1% BSA in PBST (PBS with 0.05% Tween-20) at room temperature for 1 hr. Lung homogenate, NLF, or diluted serum serially were added to the coated plates and incubated for 1 hr at room temperature. Following washes, bound IgG and IgA were detected using HRP-conjugated goat anti-hamster IgG or IgA (Arigo Biolaboratories; Hsinchu, Taiwan) or anti-mouse IgG or IgA (Invitrogen) and developed with 1-Step Ultra TMB-ELISA Substrate Solution (ThermoFisher).

### RBD-hACE2 Inhibition Assay

The inhibition of the RBD-hACE2 interaction of the hamster serum was performed using the anti-SARS-CoV-2 neutralizing antibody titer serologic assay kit (ACROBiosystems, Newark, DE; cat#TAS-K003) by following the manufacturer’s instructions.

### Neutralization Assay

The neutralizing antibody titers in hamster and mouse serum were evaluated with the VSV- and lentivirus-based pseudoviruses or with SARS-CoV-2 virus Wuhan strain. Detailed information can be found in the Supplementary Methods.

### Bioinformatics Modeling and Docking

The structures of the WT and mutated-S (R682G and S813Y) were predicted and modeled using the Phyre2 web portal (45). Molecular Docking was used to understand the binding mechanisms of S peptide substrates with Furin (PDB ID: 7HZD) and TMPRSS2 (PDB ID: 7MEQ) proteases, both in ligand-bound complex forms. To further investigate the binding mechanisms of the WT and mutant substrates with targets, we performed docking using iGEMDOCK (30). Detailed information can be found in the **Supplementary Methods**.

### Quantitative Real-Time PCR

RNA was extracted from lung homogenate using TRIzol LS (ThermoFisher) and subjected to qRT-PCR using the KAPA PROBE FAST One-Step kit (Roche, Basel, Switzerland) with primers and probes specific for the SARS-CoV-2 N and E genes, and γ-actin (housekeeping gene). To examine the cytokine responses, RNA was isolated from lung homogenates or splenocytes as described above. cDNA was synthesized using the FIREScript^®^ RT cDNA synthesis KIT (Solis BioDyne, Tartu, Estonia) and subjected to the qPCR with gene-specific primers and SYBR green dye to determine the quantification cycle (Cq). All reactions were detected using the Applied Biosystems QuantStudio 6 and 7 Flex Real-Time PCR Systems. Relative RNA expression level was calculated using the ΔΔCq method with γ-actin as an internal control. The primer sequences used in the study are listed in the Supplementary Methods.

### Immunohistochemistry

Paraffin-embedded tissue sections were rehydrated using a standard procedure and subjected to antigen retrieval with S1700 Target Retrieval Solution (Agilent, Santa Clara, CA). Anti-N antibody (GeneTex#135357) was used to stain virus-infected cells at 4°C overnight, followed by incubation with a secondary antibody (EnVision^+^ system-HRP labeled polymer, Agilent) at RT for 1 hr. The sections were then incubated with DAB substrate for color development and counterstained with hematoxylin. The integrated density of the N staining signal was further quantified by ImageJ software.

### Flow Cytometry

The spleens were collected from the vaccinated mice and the single-cell suspensions of splenocytes were prepared, washed twice with FACS buffer, and maintained in the dark at 4°C for the staining procedure. To stain intracellular cytokines, GolgiPlug™ Protein Transport Inhibitor containing Brefeldin A (BD 555029) was supplied to cell culture at 5 h before cell collection. Viable cells were gated after staining with Fixable Viability Stain 620 (564996, BD Horizon™). For FACS staining, cells were first incubated with PE-Labeled Tetramer (H-2K(b) SARS-CoV-2 S 539-546 VNFNFNGL, NIH Tetramer Core Facility), APC-CD3e (145-2C1, BD), BV421-CD8 (53-6.7, BioLegend), BV510-CD4 (RM4-5, BD) for 60 min on ice, followed by two washes in FACS buffer. The cells were fixed and permeabilized using the Cytofix/Cytoperm™ Fixation/Permeabilization Solution Kit (554714, BD Pharmingen™), and incubated with Alexa488-IFN-γ (XMG1.2, BD) for 30 min on ice. A flow cytometer (CantoII; BD) and FACSDiva software (BD) were used to acquire flow cytometric data, with FlowJo software^TM10^ used for analysis.

### Statistical Analysis

All data are denoted as the mean ± SEM and were analyzed using one-way ANOVA with Bartlett’s test *via* GraphPad Prism software. Statistical significance was assigned when *p-values* were < 0.05.

## Data Availability Statement

The raw data supporting the conclusions of this article will be made available by the authors, without undue reservation.

## Ethics Statement

All animal-related experiments were conducted in compliance with the guidelines of the Laboratory Animal Center of NHRI. The animal protocol (NHRI-IACUC-109102-A-S02 and NHRI-IACUC-109139-A) was approved by the Institutional Animal Care and Use Committee of NHRI, according to the Guide for the Care and Use of Laboratory Animals (NRC 2011).

## Author Contributions

Conduct the research work: J-JL, C-FT, Y-PK, E-JL, W-HT, M-YC, P-JT, NP, Z-SC, H-CW, W-TT, S-SD, H-CL, KMC, Y-SS. Manuscript preparation J-JL, C-FT, Y-WS, NP, G-YY. Experiment design and result discussion: Y-WS, J-MY, C-YY, T-HC, S-JL, H-WC, H-YD, F-JC, C-TC, C-LL, G-YY. All authors contributed to the article and approved the submitted version.

## Funding

The funding is provided by the Ministry of Health and Welfare, Taiwan (MOHW 109-TDU-C-222-000010), by the Ministry of Science and Technology (MOST 109-2327-B-400-004), and by NHRI (IV-111-PP-03).

## Conflict of Interest

The authors declare that the research was conducted in the absence of any commercial or financial relationships that could be construed as a potential conflict of interest.

## Publisher’s Note

All claims expressed in this article are solely those of the authors and do not necessarily represent those of their affiliated organizations, or those of the publisher, the editors and the reviewers. Any product that may be evaluated in this article, or claim that may be made by its manufacturer, is not guaranteed or endorsed by the publisher.
