# Supplementary material for: Furin and TMPRSS2 Resistant Spike Induces Robust Humoral and Cellular Immunity Against SARS-CoV-2 Lethal Infection
Source: Front Immunol. 2022 May 2;13:872047. doi: 10.3389/fimmu.2022.872047 (PMC9108258; doi:10.3389/fimmu.2022.872047)
Supplement: Supplementary file 1 [file DataSheet_1.docx]

**
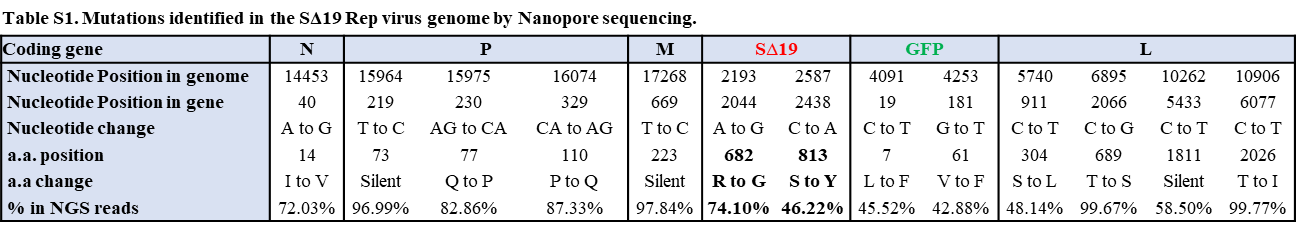
**


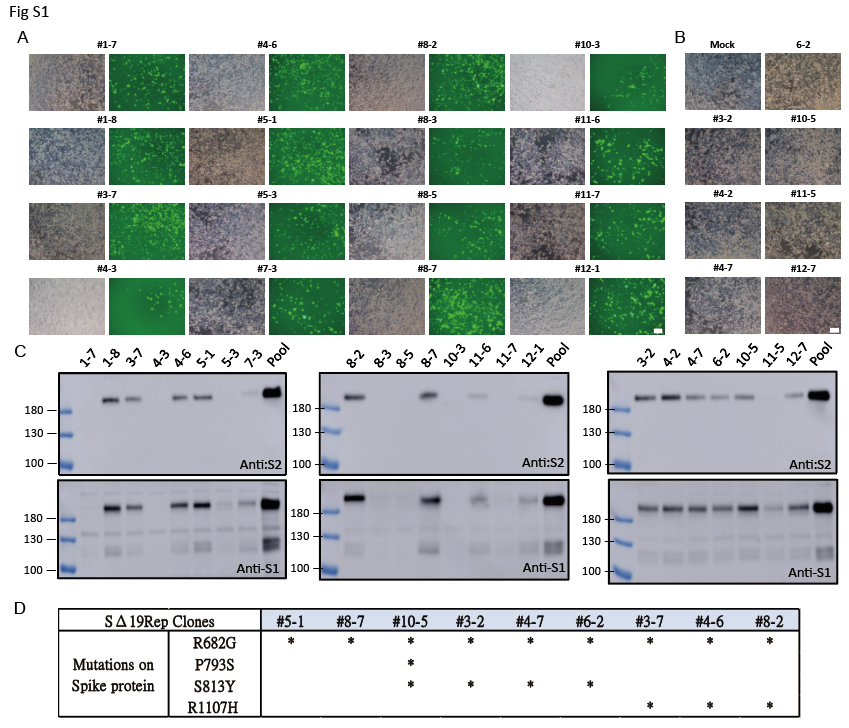


**Figure S1. Isolation of** **the S∆19 Rep clones.** The limited-diluted S∆19 Rep viruses were used to infect BHK-21/hACE2 cells and further amplified in HEK293T/hACE2 cells. The virus replication was monitored by GFP expression and cytopathic effect (CPE). (A) Photos of the clones with GFP expression. (B) The clones induced CPF in cells but without GFP expression. (C) The viral supernatant of the S∆19 Rep clones was subjected to immunoblotting to detect S protein expression. (D) The S gene sequence in the RNA genome of the S∆19 Rep clones was analyzed. The mutations of the S∆19 Rep clones were listed.


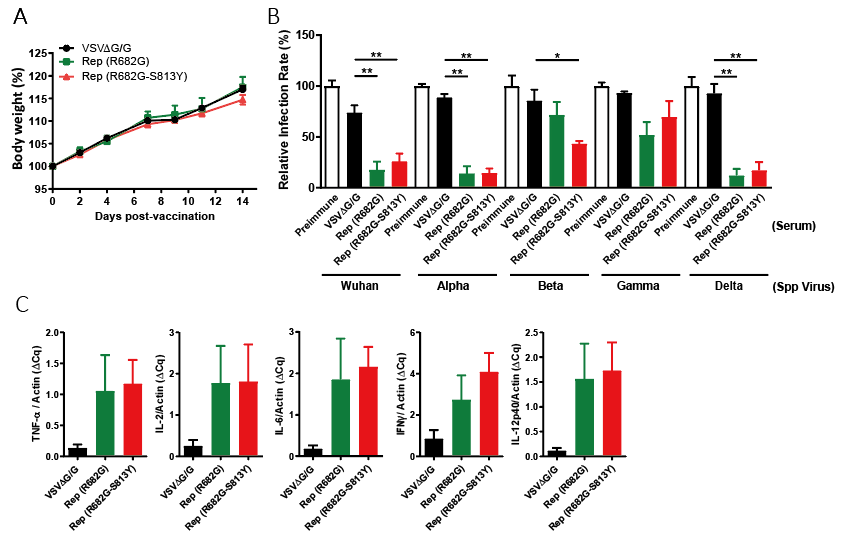


**Figure S2. The S∆19 Rep clones were used to immunize hamsters.** (A) Bodyweight of the immunized hamsters. (B) The neutralization assay of the hamster serum samples (14 days post-vaccination) with the S variant pseudoviruses (S_pp_). (C) Cytokine expression levels (TNF-α, IL-2, IL-6, IFN-γ, and IL-12p40) in the spleen from the immunized hamsters were measured by qRT-PCR. **P<*0.05; ***P<*0.01.


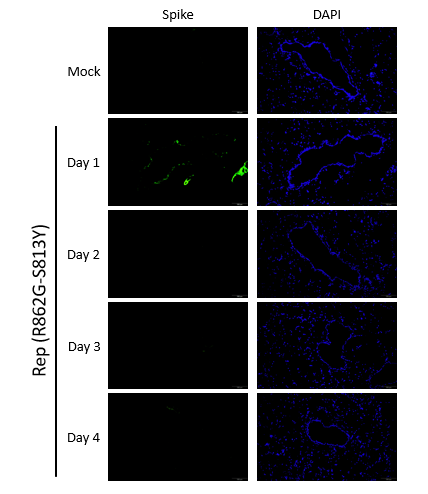


**Figure S3.** Lung sections obtained from K18-hACE2 mice infected with S∆19 Rep R682G-S813Y virus (1×10^8^ pfu or ffu/mouse; i.n.) on Day 1-4 post-infection were stained with anti-S antibodies and counterstained with DAPI.
