# Supplementary material for: Furin and TMPRSS2 Resistant Spike Induces Robust Humoral and Cellular Immunity Against SARS-CoV-2 Lethal Infection
Source: Front Immunol. 2022 May 2;13:872047. doi: 10.3389/fimmu.2022.872047 (PMC9108258; doi:10.3389/fimmu.2022.872047)
Supplement: Supplementary file 2 [file DataSheet_2.docx]

**Supplementary Methods**

**Generation of the replication-competent VSVΔG-SΔ19 (SΔ19 Rep) virus**

In Brief, HEK293T-hACE2 cells were infected with vaccinia virus T7 (VVT7) and subsequently co-transfected with VSVΔG-S∆19 and other helper plasmids (VSV N, P, L, and G) using lipofectamine 2000. Supernatants were collected at 72 hrs post-transfection, centrifuged at 1000 ×g for 5 min to remove cell debris, and filtered through a 0.1 mm filter to remove VVT7. The recovered VSVΔG-S∆19 viruses enveloped with VSV-G were used to infect the BHK21-hACE2 cells with limiting dilution in 96 well plates. The replication-competent virus (SΔ19 Rep) emerging from the infected cells was monitored by GFP expression was collected and further amplified in HEK293T-hACE2 cells at 34°C. To obtain SΔ19 Rep clones, the same limiting dilution and amplification procedure was performed again. Virus titer (pfu/ml) was performed in BHK21-hACE2 cells by tissue culture infectious dose (TCID_50_) assay. The SΔ19 Rep viral RNA in culture was were extracted and subjected to the amplicon sequencing assay utilizing the ONT MinION (28) .

**Neutralization Assay**

**1. VSV-based pseudoviruses:** Heat-inactivated serum samples (56°C, 30 min) were diluted (1:900) in 1X RPMI buffer and incubated with VSVΔG-GFP/S virus (wild type or variants) (~1.5x10^4^ ffu) for 1 h at 37°C. Serum-virus mixtures were used to infect BHK21-hACE2 cells at 37°C (n=3). After 24 incubation, infected cells were trypsinized and fixed with 4% paraformaldehyde. The viral infection rate was measured by determining the percentage of GFP-positive cells using flow cytometry (BD FACScalibur).

**2. SARS-CoV-2:** Heat-inactivated serum was serially diluted in M199 medium and mixed with SARS-CoV-2 virus (200 TCID_50_) for 2 h at 37°C. The mixture was added in quadruplicates to the cells and incubated at 37°C for 4-5 days. The neutralization titer of the serum sample was determined by the reciprocal of the highest serum dilution for which the virus infectivity is reduced by 50%.

**3. S^variant^EM-LvFLuc pseudoviruses:** The heat-inactivated serum was 1:625 diluted and mixed with S^variant^EM-LvFLuc viruses (4x10^8^ vRNA copies/assay) for 1 h at 37°C. The mixture was added to the BHK21-hACE2 cells in triplicate and incubated at 37°C for another 6 h. The relative infectivity was measured by the Luciferase Assay System (E1501, Promega) with the GLOMAX Multi^+^ Microplate Multimode Reader (Promega) at 48 hpi. and calculated by the percentage to the control groups.

**Bioinformatics modelling and docking**

The WT-S protein structure in open form trimer (PDB ID: 7DK3) was downloaded from the PDB database and used as a reference structure. The structures of the mutated-S (R682G and S813Y) were predicted and modeled using Phyre2 web portal (45). The two structure models were then aligned with the reference WT structure. Molecular Docking was used to study the binding mechanisms of S peptide substrates with Furin (PDB ID: 7HZD) and TMPRSS2 (PDB ID: 7MEQ) proteases, both in ligand bound complex forms. The structures of the Furin substrate peptide ligands PRRAR (WT) and PGRAR (mutants) were modelled from the reference peptide-mimic (ARG-ARG-ARG-LYS-ARG-00S) in 6HZD, TMPRSS2 substrate peptide ligands PSKR (WT) and PYKR (mutants) were modelled using reference 7MEQ ligand (GBS), using the Avogadro tool (Version 1.2.0) (46). To further investigate the binding mechanisms of the WT and mutant substrates with targets, we performed docking using iGEMDOCK (30). The substrate binding poses were selected based on their similarity to the crystallized ligands and the interaction profile (E-H-V protein-ligand interactions) and total Interaction energies (I.E) were calculated. The 2D structures were drawn using ChemDraw (<https://chemdrawdirect.perkinelmer.cloud/js/sample/index.html>).

**Quantitative real-time (qRT)-PCR : Primer list**

| N gene | forward: 5′-GGGGAACTTCTCCTGCTAGAAT-3′,  reverse: 5′-CAGACATTTTGCTCTCAAGCTG-3′,  probe: 5′-TTGCTGCTGCTTGACAGATT-3′ (HEK-BHQ); |
| --- | --- |
| E gene | forward: 5′-ACAGGTACGTTAATAGTTAATAGCGT-3′,  reverse: 5′-ATATTGCAGCAGTACGCACACA-3′,  probe: 5′- ACACTAGCCATCCTTACTGCGCTT CG-3′ (FAM-BHQ); |
| γ-actin | forward: 5′-ACAGAGAGAAGATGACGCAGATAAT G-3′,  reverse: 5′-GCCTGAATGGCCACGTACA-3′,  probe: 5′-TTGAAACCTTCAACACCCCAGCC-3′ (Cy5-BHQ); |
| IFN-γ | forward: 5′-GCTTAGATGTCGTGAATGG-3′,  reverse: 5′-GCTGCTGTTGAAGAAGTTAG-3′; |
| TNF-α | forward: 5′-TGAGCCATCGTGCCAATG-3′,  reverse: 5′-AGCCCGTCTGCTGGTATCAC-3′; |
| IL-2 | forward: 5′-GTGCACCCACTTCAAGCTCTAA-3′,  reverse: 5′-AAGCTCCTGTAAGTCCAGCAGTA AC-3′; |
| IL-12 | forward: 5′- GTGCACCCACTTCAAGCTCTAA-3′,  reverse: 5′- AAGCTCCTGTAAGTCCAGCAGTAAC-3′. |
| IL-6 | forward: 5′-CCTGAAAGCACTTGAAGAATTCC-3′  reverse: 5′-GGTATGCTAAGGCACAGCACACT-3′ |

**Reference**

46. Hanwell MD, Curtis DE, Lonie DC, Vandermeersch T, Zurek E, Hutchison GR. Avogadro: an advanced semantic chemical editor, visualization, and analysis platform. *J Cheminform* (2012) 4:17. doi: 10.1186/1758-2946-4-17.
