# Supplementary material for: Furin and TMPRSS2 Resistant Spike Induces Robust Humoral and Cellular Immunity Against SARS-CoV-2 Lethal Infection
Source: Front Immunol. 2022 May 2;13:872047. doi: 10.3389/fimmu.2022.872047 (PMC9108258; doi:10.3389/fimmu.2022.872047)
Supplement: Supplementary file 3 [file DataSheet_3.docx]

nCoV Spike del19

atg ttc gtg ttt ctg gtc ctg ctg cct ctg gtc tcc tct cag tgc gtg aat ctg aca acc

M F V F L V L L P L V S S Q C V N L T T

cga act cag ctg cca ccc gcc tat act aat tcc ttc acc cgg ggc gtg tac tat cct gac

R T Q L P P A Y T N S F T R G V Y Y P D

aag gtg ttt aga agc tcc gtg ctg cac tct aca cag gat ctg ttt ctg cca ttc ttt agc

K V F R S S V L H S T Q D L F L P F F S

aac gtg acc tgg ttc cac gcc atc cac gtg agc ggc acc aat ggc aca aag cgg ttc gac

N V T W F H A I H V S G T N G T K R F D

aat ccc gtg ctg cct ttt aac gat ggc gtg tac ttc gcc tct acc gag aag agc aac atc

N P V L P F N D G V Y F A S T E K S N I

atc aga ggc tgg atc ttt ggc acc aca ctg gac tcc aag aca cag tct ctg ctg atc gtg

I R G W I F G T T L D S K T Q S L L I V

aac aat gcc acc aac gtg gtc atc aag gtg tgc gag ttc cag ttt tgt aat gat ccc ttc

N N A T N V V I K V C E F Q F C N D P F

ctg ggc gtg tac tat cac aag aac aat aag agc tgg atg gag tcc gag ttt aga gtg tat

L G V Y Y H K N N K S W M E S E F R V Y

tct agc gcc aac aat tgc aca ttt gag tac gtg tcc cag cct ttc ctg atg gac ctg gag

S S A N N C T F E Y V S Q P F L M D L E

ggc aag cag ggc aat ttc aag aac ctg agg gag ttc gtg ttt aag aat atc gat ggc tac

G K Q G N F K N L R E F V F K N I D G Y

ttc aag atc tac tct aag cac acc ccc atc aac ctg gtg cgc gac ctg cct cag ggc ttc

F K I Y S K H T P I N L V R D L P Q G F

agc gcc ctg gag cca ctg gtg gat ctg cct atc ggc atc aac atc acc cgg ttt cag aca

S A L E P L V D L P I G I N I T R F Q T

ctg ctg gcc ctg cac aga agc tac ctg aca ccc ggc gac tcc tct agc gga tgg acc gca

L L A L H R S Y L T P G D S S S G W T A

gga gct gcc gcc tac tat gtg ggc tat ctg cag cct agg acc ttc ctg ctg aag tac aac

G A A A Y Y V G Y L Q P R T F L L K Y N

gag aat ggc acc atc aca gac gcc gtg gat tgc gcc ctg gat cct ctg agc gag aca aag

E N G T I T D A V D C A L D P L S E T K

tgt aca ctg aag tcc ttt acc gtg gag aag ggc atc tat cag aca tcc aat ttc agg gtg

C T L K S F T V E K G I Y Q T S N F R V

cag cca acc gag tct atc gtg cgc ttt cct aat atc aca aac ctg tgc cca ttt ggc gag

Q P T E S I V R F P N I T N L C P F G E

gtg ttc aac gca acc agg ttc gcc agc gtg tac gca tgg aat agg aag cgc atc tct aac

V F N A T R F A S V Y A W N R K R I S N

tgc gtg gcc gac tat agc gtg ctg tac aac tcc gcc tct ttc agc acc ttt aag tgc tat

C V A D Y S V L Y N S A S F S T F K C Y

ggc gtg tcc ccc aca aag ctg aat gac ctg tgc ttt acc aac gtg tac gcc gat tct ttc

G V S P T K L N D L C F T N V Y A D S F

gtg atc agg ggc gac gag gtg cgc cag atc gca cct gga cag aca ggc aag atc gcc gac

V I R G D E V R Q I A P G Q T G K I A D

tac aat tat aag ctg cca gac gat ttc acc ggc tgc gtg atc gcc tgg aac agc aac aat

Y N Y K L P D D F T G C V I A W N S N N

ctg gat tcc aaa gtg ggc ggc aac tac aat tat ctg tac cgg ctg ttt aga aag agc aat

L D S K V G G N Y N Y L Y R L F R K S N

ctg aag ccc ttc gag agg gac atc tct aca gag atc tac cag gcc ggc agc acc cct tgc

L K P F E R D I S T E I Y Q A G S T P C

aat ggc gtg gag ggc ttt aac tgt tat ttc cca ctg cag tcc tac ggc ttc cag ccc aca

N G V E G F N C Y F P L Q S Y G F Q P T

aac ggc gtg ggc tat cag cct tac cgc gtg gtg gtg ctg agc ttt gag ctg ctg cac gca

N G V G Y Q P Y R V V V L S F E L L H A

cca gca aca gtg tgc gga ccc aag aag tcc acc aat ctg gtg aag aac aag tgc gtg aac

P A T V C G P K K S T N L V K N K C V N

ttc aac ttc aac ggc ctg acc gga aca ggc gtg ctg acc gag tcc aac aag aag ttc ctg

F N F N G L T G T G V L T E S N K K F L

cca ttt cag cag ttc ggc agg gac atc gca gat acc aca gac gcc gtg cgc gac cca cag

P F Q Q F G R D I A D T T D A V R D P Q

acc ctg gag atc ctg gat atc aca ccc tgc tct ttc ggc ggc gtg agc gtg atc aca cca

T L E I L D I T P C S F G G V S V I T P

gga acc aat aca agc aac cag gtg gcc gtg ctg tat cag gac gtg aat tgt acc gag gtg

G T N T S N Q V A V L Y Q D V N C T E V

cct gtg gcc atc cac gcc gat cag ctg acc cca aca tgg cgg gtg tac agc acc ggc tcc

P V A I H A D Q L T P T W R V Y S T G S

aac gtg ttc cag aca aga gca gga tgc ctg atc gga gca gag cac gtg aac aat tcc tat

N V F Q T R A G C L I G A E H V N N S Y

gag tgc gac atc cca atc ggc gcc ggc atc tgt gcc tct tac cag acc cag aca aac tct

E C D I P I G A G I C A S Y Q T Q T N S

cca agg aga gca cgg agc gtg gcc tcc cag tct atc atc gcc tat acc atg tcc ctg ggc

P R R A R S V A S Q S I I A Y T M S L G

gcc gag aat tct gtg gcc tac tct aac aat agc atc gcc atc cca acc aac ttc aca atc

A E N S V A Y S N N S I A I P T N F T I

tct gtg acc aca gag atc ctg ccc gtg tcc atg acc aag aca tct gtg gac tgc aca atg

S V T T E I L P V S M T K T S V D C T M

tat atc tgt ggc gat tct acc gag tgc agc aac ctg ctg ctg cag tac ggc agc ttt tgt

Y I C G D S T E C S N L L L Q Y G S F C

acc cag ctg aat aga gcc ctg aca ggc atc gcc gtg gag cag gac aag aac aca cag gag

T Q L N R A L T G I A V E Q D K N T Q E

gtg ttc gcc cag gtg aag cag atc tac aag acc ccc cct atc aag gac ttt ggc ggc ttc

V F A Q V K Q I Y K T P P I K D F G G F

aac ttc agc cag atc ctg cct gat cca tcc aag ccc tct aag cgg agc ttt atc gag gac

N F S Q I L P D P S K P S K R S F I E D

ctg ctg ttc aac aag gtg acc ctg gcc gat gcc ggc ttc atc aag cag tat ggc gat tgc

L L F N K V T L A D A G F I K Q Y G D C

ctg ggc gac atc gca gca cgg gac ctg atc tgt gcc cag aag ttt aat ggc ctg acc gtg

L G D I A A R D L I C A Q K F N G L T V

ctg cca ccc ctg ctg aca gat gag atg atc gca cag tac aca agc gcc ctg ctg gcc gga

L P P L L T D E M I A Q Y T S A L L A G

acc atc aca tcc gga tgg acc ttc ggc gca gga gcc gcc ctg cag atc cct ttt gcc atg

T I T S G W T F G A G A A L Q I P F A M

cag atg gcc tat agg ttc aac ggc atc ggc gtg acc cag aat gtg ctg tac gag aac cag

Q M A Y R F N G I G V T Q N V L Y E N Q

aag ctg atc gcc aat cag ttt aac tcc gcc atc ggc aag atc cag gac agc ctg tcc tct

K L I A N Q F N S A I G K I Q D S L S S

aca gcc tcc gcc ctg ggc aag ctg cag gat gtg gtg aat cag aac gcc cag gcc ctg aat

T A S A L G K L Q D V V N Q N A Q A L N

acc ctg gtg aag cag ctg agc tcc aac ttc ggc gcc atc tct agc gtg ctg aat gat atc

T L V K Q L S S N F G A I S S V L N D I

ctg agc agg ctg gac aag gtg gag gca gag gtg cag atc gac cgg ctg atc aca ggc aga

L S R L D K V E A E V Q I D R L I T G R

ctg cag tct ctg cag acc tat gtg aca cag cag ctg atc agg gca gca gag atc agg gcc

L Q S L Q T Y V T Q Q L I R A A E I R A

agc gcc aat ctg gca gca acc aag atg tcc gag tgc gtg ctg ggc cag tct aag aga gtg

S A N L A A T K M S E C V L G Q S K R V

gac ttt tgt ggc aag ggc tat cac ctg atg tcc ttc cct cag tct gcc cca cac ggc gtg

D F C G K G Y H L M S F P Q S A P H G V

gtg ttt ctg cac gtg acc tac gtg ccc gcc cag gag aag aac ttc acc aca gcc cct gcc

V F L H V T Y V P A Q E K N F T T A P A

atc tgc cac gat ggc aag gcc cac ttt cca agg gag ggc gtg ttc gtg tcc aac ggc acc

I C H D G K A H F P R E G V F V S N G T

cac tgg ttt gtg aca cag cgc aat ttc tac gag ccc cag atc atc acc aca gac aat aca

H W F V T Q R N F Y E P Q I I T T D N T

ttc gtg tct ggc aac tgt gac gtg gtc atc ggc atc gtg aac aat acc gtg tat gat cca

F V S G N C D V V I G I V N N T V Y D P

ctg cag ccc gag ctg gac agc ttt aag gag gag ctg gat aag tac ttc aag aat cac acc

L Q P E L D S F K E E L D K Y F K N H T

tcc ccc gac gtg gat ctg ggc gac atc agc ggc atc aat gcc tcc gtg gtg aac atc cag

S P D V D L G D I S G I N A S V V N I Q

aag gag atc gac cgc ctg aac gag gtg gcc aag aat ctg aac gag tcc ctg atc gat ctg

K E I D R L N E V A K N L N E S L I D L

cag gag ctg ggc aag tat gag cag tac atc aag tgg cct tgg tac atc tgg ctg ggc ttc

Q E L G K Y E Q Y I K W P W Y I W L G F

atc gcc ggc ctg atc gcc atc gtg atg gtg acc atc atg ctg tgc tgt atg aca tcc tgc

I A G L I A I V M V T I M L C C M T S C

tgt tct tgc ctg aag ggc tgc tgt agc tgt ggc tcc tgc tgt tac ccc tat gat gtc ccc

C S C L K G C C S C G S C C Y P Y D V P

gat tac gcc tga

D Y A -

YPYDVPDYA HA epitode

R682(agg) and S813(tct) are highlighted in yellow.

nCoV Spike del19 R682G/S813Y

atg ttc gtg ttt ctg gtc ctg ctg cct ctg gtc tcc tct cag tgc gtg aat ctg aca acc

 M   F   V   F   L   V   L   L   P   L   V   S   S   Q   C   V   N   L   T   T

cga act cag ctg cca ccc gcc tat act aat tcc ttc acc cgg ggc gtg tac tat cct gac

 R   T   Q   L   P   P   A   Y   T   N   S   F   T   R   G   V   Y   Y   P   D

aag gtg ttt aga agc tcc gtg ctg cac tct aca cag gat ctg ttt ctg cca ttc ttt agc

 K   V   F   R   S   S   V   L   H   S   T   Q   D   L   F   L   P   F   F   S

aac gtg acc tgg ttc cac gcc atc cac gtg agc ggc acc aat ggc aca aag cgg ttc gac

 N   V   T   W   F   H   A   I   H   V   S   G   T   N   G   T   K   R   F   D

aat ccc gtg ctg cct ttt aac gat ggc gtg tac ttc gcc tct acc gag aag agc aac atc

 N   P   V   L   P   F   N   D   G   V   Y   F   A   S   T   E   K   S   N   I

atc aga ggc tgg atc ttt ggc acc aca ctg gac tcc aag aca cag tct ctg ctg atc gtg

 I   R   G   W   I   F   G   T   T   L   D   S   K   T   Q   S   L   L   I   V

aac aat gcc acc aac gtg gtc atc aag gtg tgc gag ttc cag ttt tgt aat gat ccc ttc

 N   N   A   T   N   V   V   I   K   V   C   E   F   Q   F   C   N   D   P   F

ctg ggc gtg tac tat cac aag aac aat aag agc tgg atg gag tcc gag ttt aga gtg tat

 L   G   V   Y   Y   H   K   N   N   K   S   W   M   E   S   E   F   R   V   Y

tct agc gcc aac aat tgc aca ttt gag tac gtg tcc cag cct ttc ctg atg gac ctg gag

 S   S   A   N   N   C   T   F   E   Y   V   S   Q   P   F   L   M   D   L   E

ggc aag cag ggc aat ttc aag aac ctg agg gag ttc gtg ttt aag aat atc gat ggc tac

 G   K   Q   G   N   F   K   N   L   R   E   F   V   F   K   N   I   D   G   Y

ttc aag atc tac tct aag cac acc ccc atc aac ctg gtg cgc gac ctg cct cag ggc ttc

 F   K   I   Y   S   K   H   T   P   I   N   L   V   R   D   L   P   Q   G   F

agc gcc ctg gag cca ctg gtg gat ctg cct atc ggc atc aac atc acc cgg ttt cag aca

 S   A   L   E   P   L   V   D   L   P   I   G   I   N   I   T   R   F   Q   T

ctg ctg gcc ctg cac aga agc tac ctg aca ccc ggc gac tcc tct agc gga tgg acc gca

 L   L   A   L   H   R   S   Y   L   T   P   G   D   S   S   S   G   W   T   A

gga gct gcc gcc tac tat gtg ggc tat ctg cag cct agg acc ttc ctg ctg aag tac aac

 G   A   A   A   Y   Y   V   G   Y   L   Q   P   R   T   F   L   L   K   Y   N

gag aat ggc acc atc aca gac gcc gtg gat tgc gcc ctg gat cct ctg agc gag aca aag

 E   N   G   T   I   T   D   A   V   D   C   A   L   D   P   L   S   E   T   K

tgt aca ctg aag tcc ttt acc gtg gag aag ggc atc tat cag aca tcc aat ttc agg gtg

 C   T   L   K   S   F   T   V   E   K   G   I   Y   Q   T   S   N   F   R   V

cag cca acc gag tct atc gtg cgc ttt cct aat atc aca aac ctg tgc cca ttt ggc gag

 Q   P   T   E   S   I   V   R   F   P   N   I   T   N   L   C   P   F   G   E

gtg ttc aac gca acc agg ttc gcc agc gtg tac gca tgg aat agg aag cgc atc tct aac

 V   F   N   A   T   R   F   A   S   V   Y   A   W   N   R   K   R   I   S   N

tgc gtg gcc gac tat agc gtg ctg tac aac tcc gcc tct ttc agc acc ttt aag tgc tat

 C   V   A   D   Y   S   V   L   Y   N   S   A   S   F   S   T   F   K   C   Y

ggc gtg tcc ccc aca aag ctg aat gac ctg tgc ttt acc aac gtg tac gcc gat tct ttc

 G   V   S   P   T   K   L   N   D   L   C   F   T   N   V   Y   A   D   S   F

gtg atc agg ggc gac gag gtg cgc cag atc gca cct gga cag aca ggc aag atc gcc gac

 V   I   R   G   D   E   V   R   Q   I   A   P   G   Q   T   G   K   I   A   D

tac aat tat aag ctg cca gac gat ttc acc ggc tgc gtg atc gcc tgg aac agc aac aat

 Y   N   Y   K   L   P   D   D   F   T   G   C   V   I   A   W   N   S   N   N

ctg gat tcc aaa gtg ggc ggc aac tac aat tat ctg tac cgg ctg ttt aga aag agc aat

 L   D   S   K   V   G   G   N   Y   N   Y   L   Y   R   L   F   R   K   S   N

ctg aag ccc ttc gag agg gac atc tct aca gag atc tac cag gcc ggc agc acc cct tgc

 L   K   P   F   E   R   D   I   S   T   E   I   Y   Q   A   G   S   T   P   C

aat ggc gtg gag ggc ttt aac tgt tat ttc cca ctg cag tcc tac ggc ttc cag ccc aca

 N   G   V   E   G   F   N   C   Y   F   P   L   Q   S   Y   G   F   Q   P   T

aac ggc gtg ggc tat cag cct tac cgc gtg gtg gtg ctg agc ttt gag ctg ctg cac gca

 N   G   V   G   Y   Q   P   Y   R   V   V   V   L   S   F   E   L   L   H   A

cca gca aca gtg tgc gga ccc aag aag tcc acc aat ctg gtg aag aac aag tgc gtg aac

 P   A   T   V   C   G   P   K   K   S   T   N   L   V   K   N   K   C   V   N

ttc aac ttc aac ggc ctg acc gga aca ggc gtg ctg acc gag tcc aac aag aag ttc ctg

 F   N   F   N   G   L   T   G   T   G   V   L   T   E   S   N   K   K   F   L

cca ttt cag cag ttc ggc agg gac atc gca gat acc aca gac gcc gtg cgc gac cca cag

 P   F   Q   Q   F   G   R   D   I   A   D   T   T   D   A   V   R   D   P   Q

acc ctg gag atc ctg gat atc aca ccc tgc tct ttc ggc ggc gtg agc gtg atc aca cca

 T   L   E   I   L   D   I   T   P   C   S   F   G   G   V   S   V   I   T   P

gga acc aat aca agc aac cag gtg gcc gtg ctg tat cag gac gtg aat tgt acc gag gtg

 G   T   N   T   S   N   Q   V   A   V   L   Y   Q   D   V   N   C   T   E   V

cct gtg gcc atc cac gcc gat cag ctg acc cca aca tgg cgg gtg tac agc acc ggc tcc

 P   V   A   I   H   A   D   Q   L   T   P   T   W   R   V   Y   S   T   G   S

aac gtg ttc cag aca aga gca gga tgc ctg atc gga gca gag cac gtg aac aat tcc tat

 N   V   F   Q   T   R   A   G   C   L   I   G   A   E   H   V   N   N   S   Y

gag tgc gac atc cca atc ggc gcc ggc atc tgt gcc tct tac cag acc cag aca aac tct

 E   C   D   I   P   I   G   A   G   I   C   A   S   Y   Q   T   Q   T   N   S

cca gga aga gca cgg agc gtg gcc tcc cag tct atc atc gcc tat acc atg tcc ctg ggc

 P   G   R   A   R   S   V   A   S   Q   S   I   I   A   Y   T   M   S   L   G

gcc gag aat tct gtg gcc tac tct aac aat agc atc gcc atc cca acc aac ttc aca atc

 A   E   N   S   V   A   Y   S   N   N   S   I   A   I   P   T   N   F   T   I

tct gtg acc aca gag atc ctg ccc gtg tcc atg acc aag aca tct gtg gac tgc aca atg

 S   V   T   T   E   I   L   P   V   S   M   T   K   T   S   V   D   C   T   M

tat atc tgt ggc gat tct acc gag tgc agc aac ctg ctg ctg cag tac ggc agc ttt tgt

 Y   I   C   G   D   S   T   E   C   S   N   L   L   L   Q   Y   G   S   F   C

acc cag ctg aat aga gcc ctg aca ggc atc gcc gtg gag cag gac aag aac aca cag gag

 T   Q   L   N   R   A   L   T   G   I   A   V   E   Q   D   K   N   T   Q   E

gtg ttc gcc cag gtg aag cag atc tac aag acc ccc cct atc aag gac ttt ggc ggc ttc

 V   F   A   Q   V   K   Q   I   Y   K   T   P   P   I   K   D   F   G   G   F

aac ttc agc cag atc ctg cct gat cca tcc aag ccc tac aag cgg agc ttt atc gag gac

 N   F   S   Q   I   L   P   D   P   S   K   P   Y   K   R   S   F   I   E   D

ctg ctg ttc aac aag gtg acc ctg gcc gat gcc ggc ttc atc aag cag tat ggc gat tgc

 L   L   F   N   K   V   T   L   A   D   A   G   F   I   K   Q   Y   G   D   C

ctg ggc gac atc gca gca cgg gac ctg atc tgt gcc cag aag ttt aat ggc ctg acc gtg

 L   G   D   I   A   A   R   D   L   I   C   A   Q   K   F   N   G   L   T   V

ctg cca ccc ctg ctg aca gat gag atg atc gca cag tac aca agc gcc ctg ctg gcc gga

 L   P   P   L   L   T   D   E   M   I   A   Q   Y   T   S   A   L   L   A   G

acc atc aca tcc gga tgg acc ttc ggc gca gga gcc gcc ctg cag atc cct ttt gcc atg

 T   I   T   S   G   W   T   F   G   A   G   A   A   L   Q   I   P   F   A   M

cag atg gcc tat agg ttc aac ggc atc ggc gtg acc cag aat gtg ctg tac gag aac cag

 Q   M   A   Y   R   F   N   G   I   G   V   T   Q   N   V   L   Y   E   N   Q

aag ctg atc gcc aat cag ttt aac tcc gcc atc ggc aag atc cag gac agc ctg tcc tct

 K   L   I   A   N   Q   F   N   S   A   I   G   K   I   Q   D   S   L   S   S

aca gcc tcc gcc ctg ggc aag ctg cag gat gtg gtg aat cag aac gcc cag gcc ctg aat

 T   A   S   A   L   G   K   L   Q   D   V   V   N   Q   N   A   Q   A   L   N

acc ctg gtg aag cag ctg agc tcc aac ttc ggc gcc atc tct agc gtg ctg aat gat atc

 T   L   V   K   Q   L   S   S   N   F   G   A   I   S   S   V   L   N   D   I

ctg agc agg ctg gac aag gtg gag gca gag gtg cag atc gac cgg ctg atc aca ggc aga

 L   S   R   L   D   K   V   E   A   E   V   Q   I   D   R   L   I   T   G   R

ctg cag tct ctg cag acc tat gtg aca cag cag ctg atc agg gca gca gag atc agg gcc

 L   Q   S   L   Q   T   Y   V   T   Q   Q   L   I   R   A   A   E   I   R   A

agc gcc aat ctg gca gca acc aag atg tcc gag tgc gtg ctg ggc cag tct aag aga gtg

 S   A   N   L   A   A   T   K   M   S   E   C   V   L   G   Q   S   K   R   V

gac ttt tgt ggc aag ggc tat cac ctg atg tcc ttc cct cag tct gcc cca cac ggc gtg

 D   F   C   G   K   G   Y   H   L   M   S   F   P   Q   S   A   P   H   G   V

gtg ttt ctg cac gtg acc tac gtg ccc gcc cag gag aag aac ttc acc aca gcc cct gcc

 V   F   L   H   V   T   Y   V   P   A   Q   E   K   N   F   T   T   A   P   A

atc tgc cac gat ggc aag gcc cac ttt cca agg gag ggc gtg ttc gtg tcc aac ggc acc

 I   C   H   D   G   K   A   H   F   P   R   E   G   V   F   V   S   N   G   T

cac tgg ttt gtg aca cag cgc aat ttc tac gag ccc cag atc atc acc aca gac aat aca

 H   W   F   V   T   Q   R   N   F   Y   E   P   Q   I   I   T   T   D   N   T

ttc gtg tct ggc aac tgt gac gtg gtc atc ggc atc gtg aac aat acc gtg tat gat cca

 F   V   S   G   N   C   D   V   V   I   G   I   V   N   N   T   V   Y   D   P

ctg cag ccc gag ctg gac agc ttt aag gag gag ctg gat aag tac ttc aag aat cac acc

 L   Q   P   E   L   D   S   F   K   E   E   L   D   K   Y   F   K   N   H   T

tcc ccc gac gtg gat ctg ggc gac atc agc ggc atc aat gcc tcc gtg gtg aac atc cag

 S   P   D   V   D   L   G   D   I   S   G   I   N   A   S   V   V   N   I   Q

aag gag atc gac cgc ctg aac gag gtg gcc aag aat ctg aac gag tcc ctg atc gat ctg

 K   E   I   D   R   L   N   E   V   A   K   N   L   N   E   S   L   I   D   L

cag gag ctg ggc aag tat gag cag tac atc aag tgg cct tgg tac atc tgg ctg ggc ttc

 Q   E   L   G   K   Y   E   Q   Y   I   K   W   P   W   Y   I   W   L   G   F

atc gcc ggc ctg atc gcc atc gtg atg gtg acc atc atg ctg tgc tgt atg aca tcc tgc

 I   A   G   L   I   A   I   V   M   V   T   I   M   L   C   C   M   T   S   C

tgt tct tgc ctg aag ggc tgc tgt agc tgt ggc tcc tgc tgt tac ccc tat gat gtc ccc

 C   S   C   L   K   G   C   C   S   C   G   S   C   C   Y   P   Y   D   V   P

gat tac gcc tga

 D   Y   A   -

YPYDVPDYA HA epitode

R682G(gga) and S813Y(tac) are highlighted in yellow.
